# Herbal SGR Formula Prevents Acute Ethanol-Induced Liver Steatosis via Inhibition of Lipogenesis and Enhancement Fatty Acid Oxidation in Mice

**DOI:** 10.1155/2015/613584

**Published:** 2015-05-25

**Authors:** Ping Qiu, Xiang Li, De-song Kong, Huan-zhou Li, Cong-cong Niu, Su-hua Pan

**Affiliations:** ^1^College of Pharmacy, Nanjing University of Chinese Medicine, Nanjing 210023, China; ^2^National First-Class Key Discipline for Traditional Chinese Medicine, Nanjing University of Chinese Medicine, Nanjing 210023, China; ^3^Zhejiang Chinese Medical University, Hangzhou 310053, China; ^4^Nanjing Municipal Hospital of TCM, Nanjing 210001, China

## Abstract

Our previous study indicated that herbal SGR formula partially attenuates ethanol-induced fatty liver, but the underlying mechanisms remain unclear. In the present study, mice were pretreated with SGR (100 and 200 mg/kg/d bw) for 30 d before being exposed to ethanol (4.8 g/kg bw). The biochemical indices and histopathological changes were examined to evaluate the protective effects and to explore potential mechanisms by investigating the adiponectin, tumor necrosis factor-*α* (TNF-*α*), peroxisome proliferators-activated receptor-*α* (PPAR-*α*), sterol regulatory element binding protein-1c (SREBP-1c), adenosine monophosphate-activated protein kinase (AMPK), and so forth. Results showed that SGR pretreatment markedly inhibited acute ethanol-induced liver steatosis, significantly reduced serum and hepatic triglyceride (TG) level, and improved classic histopathological changes. SGR suppressed the protein expression of hepatic SREBP-1c and TNF-*α* and increased adiponectin, PPAR-*α*, and AMPK phosphorylation in the liver. Meanwhile, acute toxicity tests showed that no death or toxic side effects within 14 days were observed upon oral administration of the extracts at a dose of 16 g/kg body wt. These results demonstrate that SGR could protect against acute alcohol-induced liver steatosis without any toxic side effects. Therefore, our studies provide novel molecular insights into the hepatoprotective effect of SGR formula, which may be exploited as a therapeutic agent for ethanol-induced hepatosteatosis.

## 1. Introduction

Studies in unselected heavy drinkers of alcohol suggest that 90% develop steatosis, which is the earliest and most common histopathological manifestation of ALD [1]. It occurs in most people consuming alcohol in excess of 80 g/day and can resolve within 2–4 weeks of abstinence [2]. Alcoholic fatty liver diseases are potentially pathological conditions that can progress to steatohepatitis, fibrosis, and cirrhosis. Ethanol induces hepatic steatosis via a complex mechanism that is not clear and requires further investigation. Early studies indicated that alcohol increases the ratio of reduced NADH/NAD^+^ in hepatocytes, which disrupts shift of the redox state of the liver and inhibits fatty acid *β*-oxidation has been documented in steatohepatitis [3].

However, the metabolic explanation for fatty liver has been a prevailing concept for many years, but it became insufficient to account for all changes that occurred in hepatic lipids accumulation after ethanol consumption. Therefore, recent studies indicate that alcohol exposure regulates specific lipid metabolism-associated transcription factors that have been proposed to participate in this mechanism. For instance, ethanol enhances fatty acid synthesis in hepatocytes through the upregulation of sterol regulatory element binding protein 1c (SREBP-1c) protein expression, which induces a battery of lipogenic enzymes [4]. In addition, alcohol promotes lipid accumulation by inhibiting peroxisome proliferator-activated receptor-*α* (PPAR-*α*) and adenosine monophosphate-activated protein kinase (AMPK), which have been investigated to control the transcription of a range of genes involved in free fatty acid transport and oxidation [5, 6]. Moreover, Gao reported that adiponectin and tumor necrosis factor-*α* (TNF-*α*) suppress mutual gene expression and antagonize their biological effects in the liver tissues [7, 8]. Available evidence suggests that adiponectin exerts a protective effect against alcoholic liver steatosis by coordinating multiple signaling pathways mediated by AMPK, PPAR-*α*, and SREBP-1c, resulting in a reduced lipogenesis [9]. However, in mice, TNF-*α* expression has been shown to enhance hepatic lipogenesis and blockage of its receptor (TNF-*α* R1) can prevent the development of alcoholic fatty liver [10]. Thus, we have summarized and integrated the new emerging factors into a schematic diagram (Figure 7) that depicts the major pathways of ethanol-elicited hepatic lipid accumulation.

Recently, an extremely popular therapeutic option for the prevention of alcohol-induced liver steatosis could resort to natural plants that are rich in flavonoids. In this study, SGR is an attractive herbal formula that is based on the principle of Traditional Chinese Medicine Theory of spleen invigoration and liver-qi depression relief, promotion of blood circulation. Moreover, SGR was indeed demonstrated to prevent alcoholic liver injury in our experimental research which contains Semen Hoveniae extract (SHE),* Ginkgo biloba* extract (GBE), and* Rosa roxburghii* Tratt extract (RRE). In particular, Semen Hoveniae, the seed of the* Hovenia dulcis* Thunb., is widely used in Chinese traditional medicine since ancient times and is thought to be nature's gift to accelerate detoxification of ethanol and possess antioxidative, antimicrobial, and antidiabetic properties [11]. GBE and RRE, both in vitro and in vivo studies have demonstrated the protective effects on the response to oxidative stress and ethanol-induced fatty liver [12, 13]. Nevertheless, the underlying molecular mechanisms of herbal SGR formula are still not fully understood. The current study was designed to evaluate the protective effects of SGR against ethanol-induced steatosis and to investigate the effects of ethanol and SGR on the levels of adiponectin, TNF-*α*, AMPK, PPAR-*α*, and SREBP-1c for the underlying mechanisms exploration using mice as an experimental model.

## 2. Materials and Methods

### 2.1. Materials and Chemicals

HPLC grade methanol was purchased from Merck (Germany). Water was purified by a Milli-Q water purification system (Millipore, USA). Aqueous phosphoric acid and ethanol of analytical grade were purchased from Guangfu Technology Co., Ltd (Tianjin, China). Standards including dihydromyricetin, dihydroquercetin, and quercetin (internal standard, IS) were purchased from the Nanjing Zelang Medical Technology Limited Company (Nanjing, China). The kit of triglyceride (TG) was the product of Kehua Co., Ltd (Shanghai, China). All the three reference compounds have over 98% purity. Primary antibodies of TNF-*α*, AMPK, P-AMPK, PPAR-*α* antibody (1 : 1000), anti-mouse (1 : 10000), and anti-rabbit (1 : 10000) secondary IgG were provided by bioworld (Nanjing, China). Primary SREBP-1c and *α*-Tublin antibody (1 : 3000) was obtained from Affinity BioReagents (Golden, CO, USA. AF7010, USA). Western blotting detecting reagents (enhanced chemiluminescence) was provided by Millipore Corp. (Billerica, MA01821, USA). BCA protein assay kits were purchased from Pierce Biotechnology, Inc. (Rockford, IL61105, USA). Adiponectin and AMPK enzyme linked immunosorbent assay (ELISA) kit was purchased from Shanghai Yuanye Biotech Industry Co., Ltd (Shanghai, China).

### 2.2. Plant Material

*H. dulcis* seeds have long been used in ancient Chinese traditional herbal medicine for the treatment of liver diseases and detoxification after alcoholic poisoning. Semen Hoveniae was harvested and obtained from Hangzhou City, Zhejiang Province, China, in November 2012 and was identified as* H. dulcis* seeds by Professor Hui Yan, who has been engaged in botanical research for several years, particularly in classifying and identifying traditional Chinese herbs. A voucher specimen (number 20121208) was kept at the College of Pharmacy, Nanjing University of Chinese Medicine.* G. biloba* and* R. roxburghii* Tratt extracts were purchased from Xinyuan Bio-Products Co., Ltd. (Pizhou, number 20091116) and Tianjiang Pharmaceutical Co., Ltd. (Jiangsu, number 20091112), respectively.

### 2.3. Preparation of SGR

The dried seeds (2 kg) were pulverized in a motor-driven grinder into coarse powder at high speed. The powder was extracted exhaustively with distilled water (1 : 8, w/v) for 2 h, water (1 : 6, w/v) for 1 h, and water (1 : 6, w/v) for 1 h by refluxing extraction method, respectively. The thrice-extracted liquids were blended, and then the obtained extracts were filtered by eight-layer gauzes and evaporated under reduced pressure on a rotary evaporator (temperature at 60–75°C) and vacuum freeze-dried. SHE milled into fine powder, which mixed with* Ginkgo biloba* extract (GBE) and* Rosa roxburghii* Tratt extract (RRTE) power at a ratio of 8 : 1 : 1 in this study (equal to the proportion of three herb extracts in SGR). The herbal SGR formula was stored at 4°C for dilution into the needed concentration in a volumetric flask.

### 2.4. Chromatographic Analysis of SGR

The flavonoids of the herbal SGR formula were analysed by HPLC. Chromatographic separation was performed on a Waters 2545 quaternary gradient module (Waters Corp., MA, USA) coupled with 2998 photodiode array (PDA) detector and equipped with an online degasser. An autosampler was used for solvent delivery. The measurements were performed on a SunFire analytical C18 column (250 mm × 4.6 mm, Waters, USA) at a column temperature of 25°C. The solvent system was a gradient of solvent A (aqueous phosphoric acid, 0.2%) and solvent B (methanol): initial composition, 78% B; linear gradient to 30% B from 0 min to 40 min; initial composition, 22% A; linear gradient to 70% B from 0 min to 40 min; and washing and reconditioning the column. The injection volume was 20 *μ*L for each sample solution. The elution was run at a flow rate of 1 mL/min, and the UV spectra were monitored in the range of 200 nm to 400 nm. The HPLC chromatogram wavelength of 290 nm was selected for the quantitative analysis of SGR.

### 2.5. Acute Toxicity Studies

The acute toxicity of SGR was performed according to the method described previously [14] with slight modification. Two groups (male and female) of ICR mice each weighing 22 ± 2 g were used (*n* = 12 for each group). SGR (16 g/kg body wt.) was orally administered and the general behavior and toxic symptoms were observed at the first critical 4 h. All mice were further kept under the same conditions for any signs of toxicity and mortality for a time span of 14 days after the treatment.

### 2.6. Animal Treatment of Acute Ethanol-Induced Liver Injury

Forty-eight specific pathogen-free male ICR mice weighing 18–22 g were purchased from Shanghai Si-Lai-Ke Experimental Animal Ltd. (Shanghai China). All mice were maintained at approximately 22 ± 2°C on a reverse 12 h light/dark cycle and had free access to commercial standard chow and tap water. Total mice were randomized into 4 groups (*n* = 12) after 3 d of acclimation to the laboratory environment. The SGR group was orally pretreated with SGR (100, 200 mg/kg) for 30 d, while the control and ethanol groups received an equal volume of saline. The animals in the SGR and ethanol groups were exposed to ethanol (4.8 g/kg, bw) 2 h after the last dosing, whereas the control group mice received an equivalent amount of saline. The mice were sacrificed after fasting for 12 h. The serum was prepared from blood by centrifugation at 3500 rpm for 10 min at 4°C (Beckman, Germany). The liver tissue was dissected rapidly and weighed. Portion of the liver was sliced and fixed in formaldehyde saline (4%) solution for the histopathological examination. The remaining liver was snap-frozen in liquid nitrogen and stored at −80°C for homogenate preparation. All experimental procedures were conducted in accordance with the National Institutes of Health Guidelines for the Care and Use of Animals and were approved by the local ethics committee.

### 2.7. Assay of the Serum and Hepatic TG Levels

The serum TG level was determined by using standard clinical chemistry assays on an Automated Chemistry Analyzer (VITROS 950, USA). To assess the liver steatosis, TG levels in the liver were extracted following the method described by Zeng et al. [15]. Briefly, portion of liver was homogenized in an equal volume of normal saline and then extracted with methanol : chloroform (1 : 2) for about 24 h. Then the hepatic TG level was quantified by automatic biochemical analyzer (AU 640 Medical System, OlymPus, Japan) according to the instructions provided by the commercial assay kits.

### 2.8. Liver Morphological and Histopathological Evaluation

Liver index (liver weight/body weight, %) was obtained by dividing liver weight in mice. Liver tissues were removed immediately and fixed in 4% neutral formalin solution for at least 24 h, then embedded in paraffin wax, sectioned (4 *μ*m thickness), stained with haematoxylin-eosin (H&E) for microscopic examination, and then analysed by a registered pathologist, who was blinded to the experimental design and the treatment.

### 2.9. Serum Adiponectin and Hepatic AMPK Determination by ELISA

The separated serum was used for analysis of adiponectin levels. AMPK were measured in 100 mg of liver that was homogenized in 1 mL 1 × PBS. And then samples were centrifuged twice at 15,000 rpm for 15 min at 4°C. The resulting supernatants were used for the assay. Adiponectin, AMPK levels were detected by a fluorescence reader using a mouse ELISA kit and were expressed as pg/mL of liver.

### 2.10. Preparation of Total Protein Extracts

The total protein extracts of mice liver tissues were prepared using RIPA buffer (50 mM Tris–HCl, 150 mM NaCl, 1% Triton X-100, 1% sodium deoxychlolate, 0.1% sodium dodecyl sulfate, 1 mM phenylmethylsulfonyl fluoride (PMSF), 1 mM Na_3_VO_4_, 5 mM NaF, and 1% cocktail protein inhibitors, pH 8.0). The homogenate was centrifuged at 15,000 rpm for 15 min, and the supernatant was kept as the total protein extract at −80°C for Western blot analysis. Protein concentrations were quantified using the BCA protein assay kit. All the above procedures were performed at 4°C.

### 2.11. Western Blot Analysis

Protein samples were mixed with 3x loading buffer and then heated at 100°C for 10 min. The pretreated protein samples (about 50 *μ*g) were separated by electrophoresis in a 10% or 12% denatured polyacrylamide gel and then transferred to a 0.22 *μ*m polyvinylidene fluoride membrane (PVDF, Bio-Rad, Germany) by gel electrophoresis and wet transfer system (Bio-Rad, Germany), respectively. After blocking for 1 h in 5% (w/v) nonfat dry milk in TBS-Tween 20 (20 mM/L Tris-Cl (pH 7.5), 137 mM/L NaCl, 0.05% (w/v) Tween-20), the membranes were exposed overnight to the primary antibody at 4°C. The membranes were washed thrice with TBST for 10 min each and then incubated with the corresponding horseradish peroxidase- (HRP-) conjugated secondary antibody at room temperature for 2 h. The membranes were then washed for 4 times in TBST for 10 min each and the proteins were visualized using an enhanced chemiluminescence (ECL) Western blotting detection reagent. The results were normalized by *α*-Tublin to ensure equal loading.

### 2.12. Immunofluorescence of PPAR-*α* in Liver

For immunofluorescent labeling, cryostat sections of liver were cut into 10 *μ*m thick sections and incubated with the primary antibody rabbit PPAR-*α* and the secondary antibody goat anti-rabbit IgG-FITC (Boshide, Wuhan, China). The experiment was performed following the manufacturer's instructions and was observed and photographed under a laser scanning confocal microscope (LEICA, Mannheim, Germany). Immunohistochemical staining of PPAR-*α* was digitally quantified from the acquired images using IPP6.0 software (IOD = integrated optical density). Average number of fluorescence dots of three images from each treatment group was calculated.

### 2.13. Statistical Analysis

Data are expressed as means ± SD, and all statistical comparisons were subjected to SPSS17.0 software. Statistical analysis was determined by one-way analysis of variance (ANOVA) followed by the post hoc Dunnett's test. A probability value of *P* < 0.05 was considered to be statistically significant.

## 3. Results

### 3.1. Preliminary Characterisation of SGR

**Figure 1:**
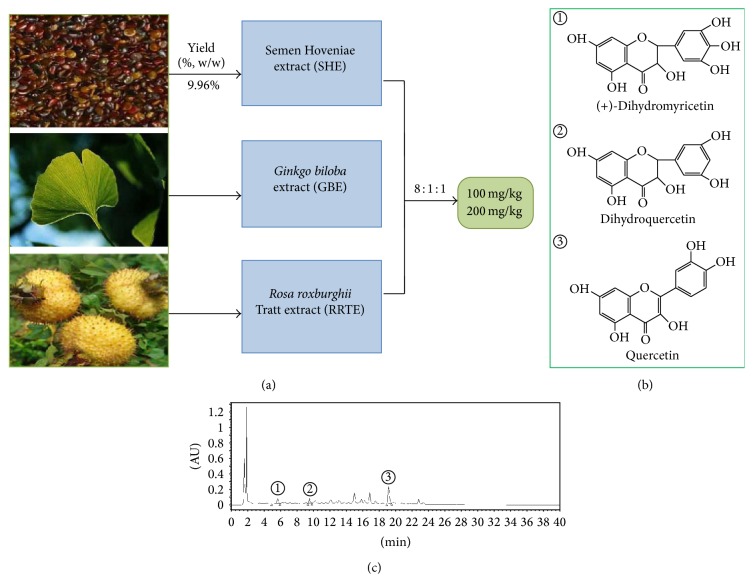
(a) Preparation of SGR, Semen Hoveniae extract (SHE) power mixed properly with* Ginkgo biloba *extract (GBE) and* Rosa roxburghii *Tratt extract (RRTE) power at the ratio of 8 : 1 : 1 in this study. (b) and (c) Flavonoids of herbal formula SGR were analysed by HPLC. ①: dihydromyricetin, ②: dihydroquercetin, and ③: quercetin.

SGR was prepared from the hot water extraction of* H. dulcis* seeds. The overall yield of SHE was 9.98% based on the dried seeds used. SHE, GBE, and RRTE were mixed at a ratio of 8 : 1 : 1 (Preparation of SGR, Figure 1(a)). The results of chemical analysis showed that SGR contained flavonoids, including 1.5% dihydromyricetin, 1% dihydroquercetin, and 11.21% quercetin, as calculated by comparing the retention times with those of the standards on HPLC (Figure 1(b)).

### 3.2. Evaluation of Acute Toxicity in Mice

Acute toxicity study was carried out for SGR to confirm safety. When SGR was studied by oral administration in mice, no signs of toxicity were observed. Moreover, our results have shown that no death or toxic side effects within 14 days were observed with oral administration of the extracts at a dose of 16 g/kg body wt.

### 3.3. Body Weight and Liver Weight Changes after SGR Feeding

**Figure 2:**
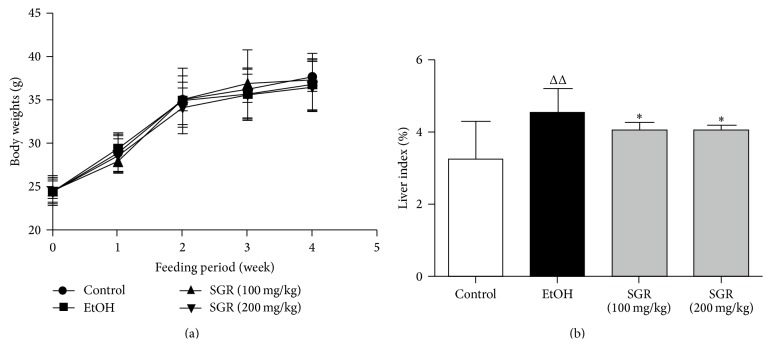
Effects of SGR on the body weight and liver index in acutely inebriated mice (*n* = 12). ^Δ^ *P* < 0.05, ^ΔΔ^ *P* < 0.01, compared with normal control group; ^*∗*^ *P* < 0.05, ^*∗∗*^ *P* < 0.01, compared with ethanol group.

Based on the preliminary studies as well as the published studies, there was no difference in body weight among all groups, and SGR supplementation did not affect the overall body weight (Figure 2(a)). Based on the preliminary studies as well as the published studies, thus, with the dose of ethanol (4.8 g/kg bw) mice were sluggish and unconscious but regained normal behavior within several hours. In ethanol group, the liver index (the ratio of liver weight to the body weight) was significantly larger than those in the control group (*P* < 0.01, Figure 2(b)). However, the data clearly showed that liver index was significantly decreased after pretreatment with SGR (100, 200 mg/kg/day bw) for 30 d (*P* < 0.05, Figure 2(b)).

### 3.4. SGR Decreases TG Levels in Serum and Liver Tissue as well as Hepatic Pathological Changes

**Figure 3:**
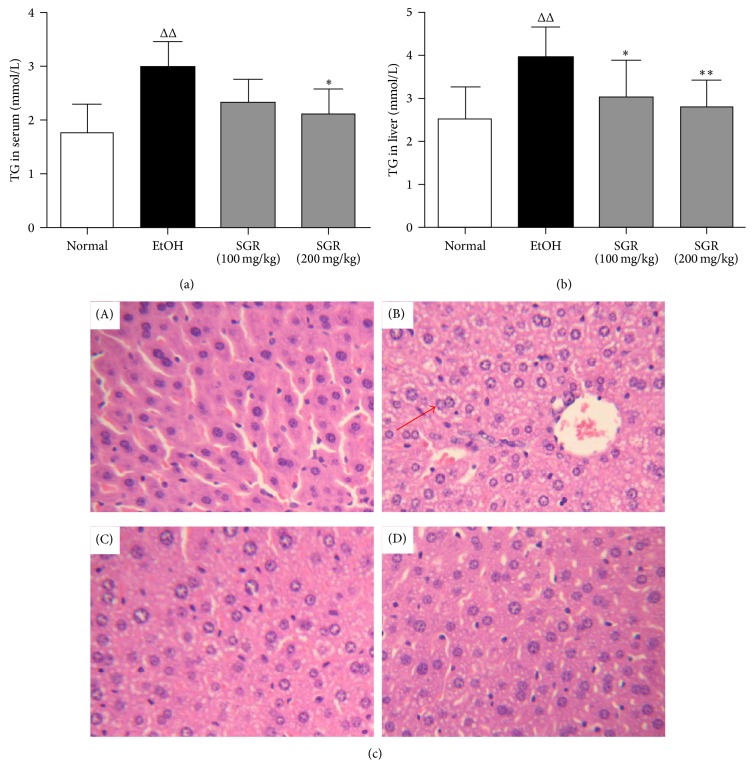
Effect of SGR pretreatment on the TG levels in serum and hepatic, pathological changes in the liver with acute alcoholism (×100). (a) and (b) Supplement with SGR for 30 d significantly inhibited the increase of the TG levels in serum and in liver, respectively. (c) H&E (hematoxylin and eosin) staining of liver tissue. The red arrow indicated the fat droplets in the liver sections. (A) normal; (B) ethanol; (C) SGR (100 mg/kg); (D) SGR (200 mg/kg). ^Δ^ *P* < 0.05, ^ΔΔ^ *P* < 0.01, compared with normal control group; ^*∗*^ *P* < 0.05, ^*∗∗*^ *P* < 0.01, compared with ethanol group.

In ethanol group, triglyceride levels in serum and liver tissue were significantly larger than those in the control group (*P* < 0.01, Figures 3(a) and 3(b)). However, the data clearly showed that TG levels in serum and liver were significantly decreased after pretreatment with SGR (100, 200 mg/kg/day bw) for 4 weeks (*P* < 0.05 or *P* < 0.01, Figures 3(a) and 3(b)). The morphological analysis showed that alcohol administration promoted microvesicular steatosis compared to the controls, as indicated by the appearance of lipid droplets (Figure 3(c) (B)). However, SGR pretreatment remarkably reduced the steatosis compared to the ethanol group (Figure 3(c) (C) and (D)).

### 3.5. Effects of SGR on Serum Adiponectin and Hepatic TNF-*α* in Acute Ethanol-Induced Mice

**Figure 4:**
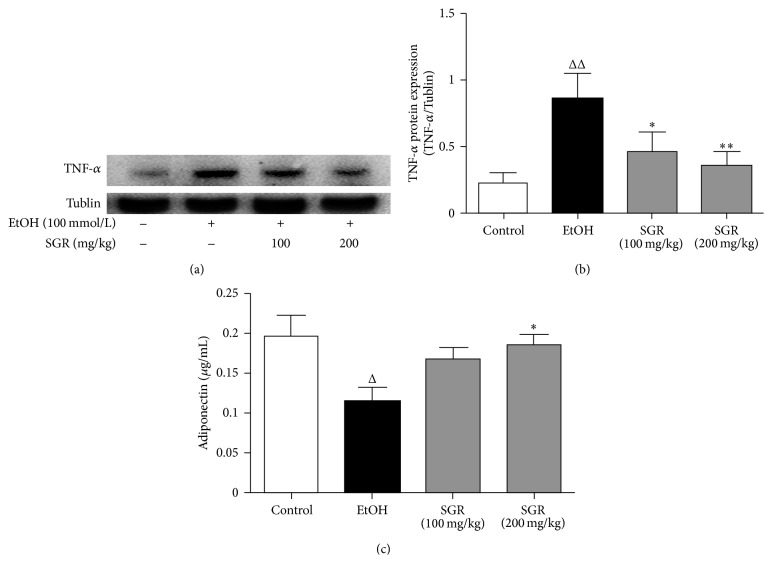
Effects of SGR on the hepatic TNF-*α* protein expression, serum adiponectin level in mice with acute ethanol exposure. (a) and (b) TNF-*α* protein levels were quantified with Tublin as an internal control and expressed as the relative content of the control value. These data were representative of three independent experiments. (c) Serum adiponectin levels were quantified with ELISA (*n* = 12). ^Δ^ *P* < 0.05, ^ΔΔ^ *P* < 0.01, compared with normal control group; ^*∗*^ *P* < 0.05, ^*∗∗*^ *P* < 0.01, compared with ethanol group.

Adiponectin and TNF-*α* play important roles in the development of liver steatosis. Therefore, changes in serum and liver cytokines of the ethanol-treated mice were examined.As shown in Figure 4, level of proinflammatory cytokine TNF-*α* in liver was found elevated significantly, whereas the level of adiponectin in serum remarkably attenuated in ethanol treated group in comparison with the control (*P* < 0.05 or *P* < 0.01, Figures 4(b) and 4(c)). The administration of SGR can significantly ameliorate the hepatic TNF-*α* level and increase the serum adiponectin level in ethanol-exposed mice (*P* < 0.05 or *P* < 0.01, Figures 4(b) and 4(c)).

### 3.6. Effects of SGR on the Protein Levels of AMPK, Phosphorylation of AMPK, PPAR-*α*, and SREBP-1c in Acute Ethanol-Induced Mice

**Figure 5:**
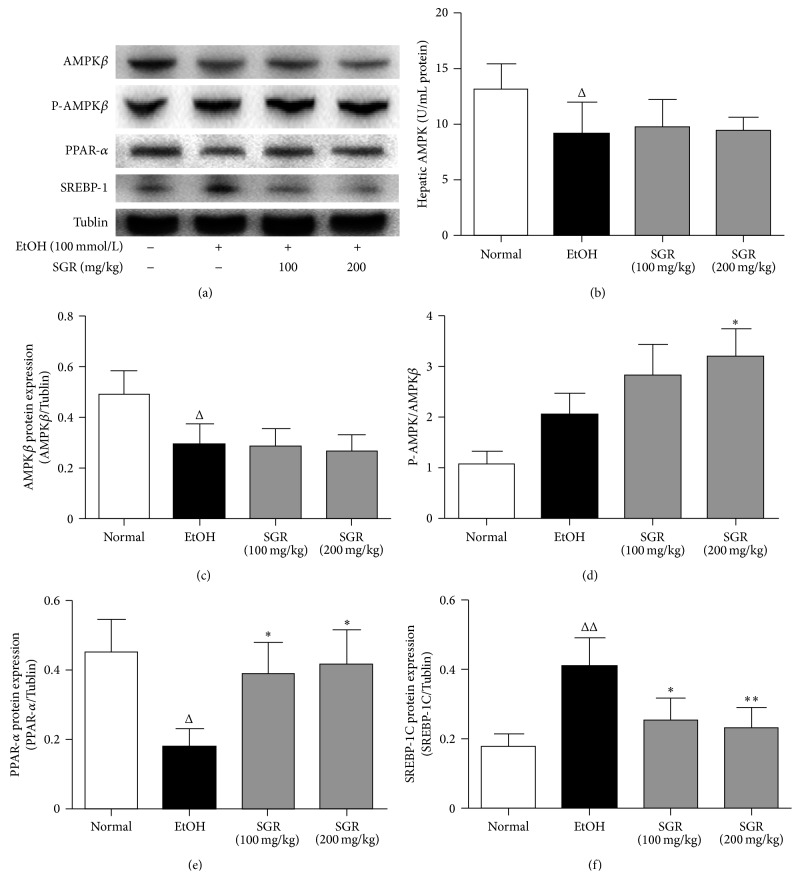
Effects of SGR on the hepatic AMPK, P-AMPK*β*, PPAR-*α*, and SREBP-1c protein expression in mice with acute ethanol exposure, respectively. (a) AMPK, P-AMPK*β*, PPAR-*α*, and SREBP-1c were quantified with Tublin as an internal control. (b) Hepatic AMPK levels were quantified with ELISA (*n* = 12). (c, e, and f) AMPK, PPAR-*α*, and SREBP-1c protein levels of them were expressed as the relative content of the control value. (d) P-AMPK*β*/AMPK expressed as the degree of phosphorylated AMPK. These data were representative of three independent experiments. ^Δ^ *P* < 0.05, ^ΔΔ^ *P* < 0.01, compared with normal control group; ^*∗*^ *P* < 0.05, ^*∗∗*^ *P* < 0.01, compared with ethanol group.

AMPK plays a central role in the regulation of lipid metabolism by inhibiting regulatory enzymes involved in biosynthetic pathways. We examined whether SGR plays a role in the development of alcohol-induced lipid synthesis by regulating the activation of AMPK in liver of animals by Western blot analysis and ELISA. However, as shown in Figure 5(a), after acute alcohol ingestion the hepatic AMPK levels in the ethanol group were attenuated significantly compared to those in the control group (*P* < 0.05, Figures 5(b) and 5(c)). In addition, our data of this study suggested that SGR (100, 200 mg/kg/day bw) did not have an apparent effect on the levels of AMPK compared to those in the ethanol group (*P* > 0.05, Figures 5(b) and 5(c)). However, we have observed that acute alcohol ingestion had positive effect on hepatic AMPK phosphorylation. In particular, SGR (200 mg/kg/day bw) can significantly elevate the phosphorylation status of AMPK compared to ethanol group (*P* < 0.05, Figure 5(d)). Meanwhile, the levels of protein expression for PPAR-*α* and the lipogenic enzymes SREPB-1c were measured by Western blot analysis to investigate whether liver steatosis in ethanol-induced mice was caused by inhibition of PPAR-*α* and activation of SREBP-1c. The protein levels of SREBP-1c in ethanol group (4.8 g/kg bw) were elevated significantly, whereas the level of PPAR-*α* in liver remarkably attenuated in ethanol treated group in comparison with the control (*P* < 0.05 or *P* < 0.01, Figures 5(e) and 5(f)). In contrast, the administration of SGR can significantly ameliorate the level of SREBP-1c and increase the level of PPAR-*α* in acute ethanol-exposed mice (*P* < 0.05 or *P* < 0.01, Figures 5(e) and 5(f)).

### 3.7. Effects of SGR on the PPAR-*α* in Acute Ethanol-Induced Mice via Immunofluorescence Staining

**Figure 6:**
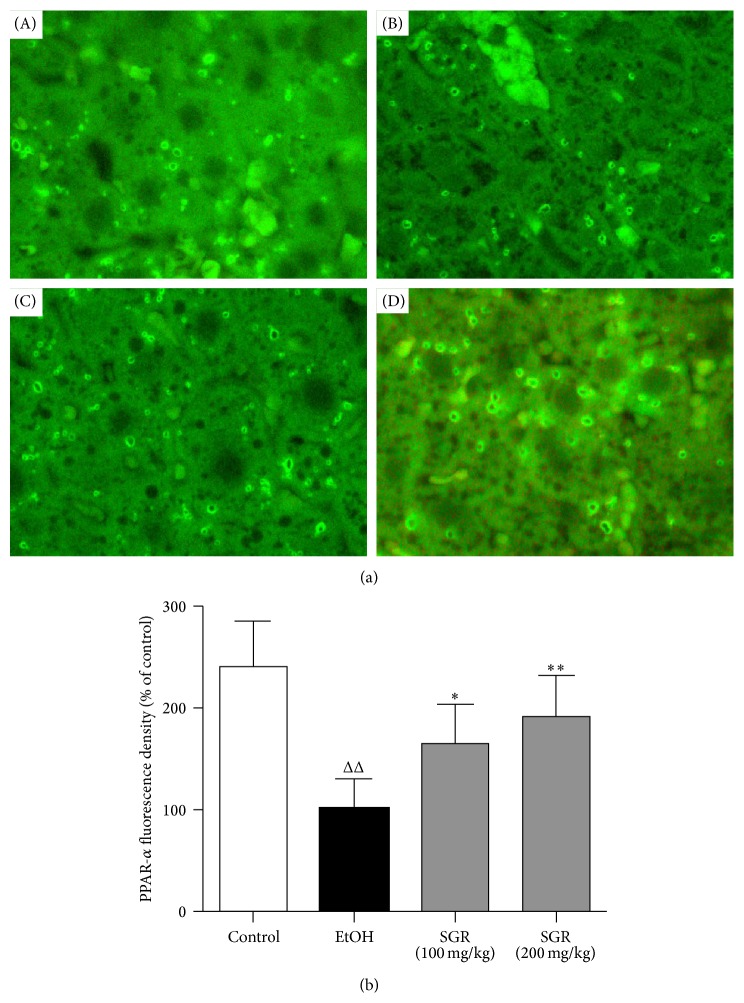
The effects of SGR supplementation on PPAR-*α* immunofluorescence staining of acute ethanol-induced liver injury in mice. The graph showed the average number of fluorescence dots of images from each treatment group (×400). (a) (A) normal; (B) ethanol; (C) SGR (100 mg/kg); (D) SGR (200 mg/kg). (b) Changes in the cumulative value of optical density in cytoplasm immunofluorescence staining of PPAR-*α*, expressed as the relative content of the ethanol control value. These data were representative of three independent experiments. ^Δ^ *P* < 0.05, ^ΔΔ^ *P* < 0.01, compared with normal control group; ^*∗*^ *P* < 0.05, ^*∗∗*^ *P* < 0.01, compared with ethanol group.

The effects of SGR on the PPAR-*α* were examined through immunofluorescence staining analysis. As shown in Figure 6, the green fluorescence density of PPAR-*α* in the hepatic cytoplasm was significantly attenuated in the ethanol group compared to the control group (Figures 6(a) (A and B) and 6(b)). However, the pretreatment with SGR at doses of 100 mg/kg and 200 mg/kg BW significantly increased the green fluorescence density of PPAR-*α* in the hepatocellular cytoplasm in a dose-dependent manner compared with those in the ethanol group (Figures 6(a) (C and D) and 6(b)). Taken together, These data combined with the Western blot results, strongly support that SGR elevated significantly PPAR-*α* protein expression to inhibit ethanol-induced liver steatosis.

## 4. Discussion

Alcoholic liver disease is associated with a state of hepatic fatty acid overload. Recent studies indicate that additional effects of ethanol impair fat oxidation and stimulate lipogenesis. The development of effective agents to prevent or treat alcohol-induced hepatic steatosis will depend on elucidating the suppression and blockage of any of the steps culminating into liver injury. For instance, ethanol metabolism in liver, SREBP 1c, and TNF-*α* are responsible for free fatty acid (FFA) synthesis, while adiponectin, AMPK, and PPAR-*α* are responsible for FFA oxidation. However, the gathered pieces of evidence suggest that the “one target one drug paradigm” in drug discovery is now facing an unprecedented challenge [16].

In recent decades, herbal medicines and their active compounds have been drawn experimental and clinical attentions for a long time due to its promising lipid-lowering effects and with the multitargeted and less toxic features were as the potential agents in the prevention of ethanol-induced fatty liver [17]. For instance, Honokiol [18], green tea [19], resveratrol [20], quercetin [21], and so forth have been applied to suppress lipid synthesis and increase of fatty acid *β*-oxidation. In this study, flavonoids of herbal formula SGR were analysed by HPLC that are rich in dihydromyricetin, dihydroquercetin, and quercetin. Furthermore, the results have demonstrated that pretreatment of SGR before ethanol intake suppresses acute ethanol-induced hepatic lipid accumulation. Moreover, acute toxicity study was carried out for SGR to confirm safety. Our results have shown that no death or acute toxicity within 14 days was observed upon oral administration of the extracts at a dose of 16 g/kg body wt.

Ethanol metabolism-associated TG accumulating can result in the development of fatty liver [7]. In the present study, supplementation of SGR against ethanol-induced steatosis markedly improved as evidenced by normalization of liver enzymes along with a significant reduction of liver index, TG level in serum and liver. Furthermore, ethanol exposure dramatically increased the hepatic fat accumulation shown by histopathological changes. Therefore, SGR treatment could ameliorate acute ethanol-induced liver damage to a high degree.

Adiponectin is an adipokine with anti-inflammatory, insulin sensitization and antifibrotic properties [22, 23]. Moreover, adiponectin is largely mediated by an increase in fatty acid oxidation, associated with activation of AMPK and PPAR-*α* pathways and suppressing hepatic production of TNF-*α* that appears to protect the liver from steatosis [24, 25]. Furthermore, available evidence now consistently demonstrates that chronic, heavy ethanol exposure decreases serum adiponectin concentrations in mice [26, 27] and rats [8]. Additionally, emerging evidence indicates that adiponectin has a key role in inactivation of TNF-*α* due to the fact that they regulate the mutual synthesis on the target tissues [28]. Compared to the well-established role of TNF-*α* in alcoholic liver inflammation and cytotoxic, its contribution in ethanol-induced steatosis was not widely characterized. Furthermore, TNF-*α* expression has been shown to increase in alcoholic fatty liver, whereas several evidence was previously reported that TNFR1 knockout almost completely blocks development of ethanol-induced fatty liver [29]. In addition, TNF-*α* has been shown to upregulate mRNA expression of SREBP-1c in the liver of mice [30] and to stimulate the maturation of SREBP-1c protein in human hepatocytes [31]. In this study, we confirmed that acute ethanol exposure resulted in the enhancement of TNF-*α* level in liver and attenuation of the serum adiponectin concentration. However, our results demonstrated that SGR can ameliorate the level of TNF-*α* and increase the level of adiponectin in acute ethanol-exposed mice, which can attenuate the expression of fatty acid synthesis proteins and enhance fatty acid oxidation.

AMPK is known to act as a key metabolic “master switch” by phosphorylating the target enzymes involved in lipid metabolism in many tissues including the liver. Steatosis induced by chronic alcohol consumption can be directly linked to a critical signaling pathway that increases lipogenesis in the liver via AMPK inhibition. Furthermore, pharmacological AMPK activation abrogated ethanol-induced induction of lipogenesis and enhancement of fatty acid *β*-oxidation [32], such as adiponectin and other agents. Interestingly, recent studies have demonstrated that AMPK inhibition by alcohol appears to be a paradox, because ethanol-mediated ROS production which is critical controlling factors in the activation of AMPK likely via different mechanisms and at different signaling pathways [33]. Paradoxically, our findings of this study suggested that acute alcohol exposure elevated the ratio of p-AMPK/AMPK compared to the control group. Nevertheless, SGR (200 mg/kg/day bw) can boost significantly the phosphorylation status of AMPK compared to the ethanol group. The results have demonstrated that SGR formula can mediate AMPK signaling to prevent acute alcohol-induced fatty liver. Interestingly, these results further confirmed the hypothesis that ROS induced by acute ethanol ingestion stimulates AMPK activity via an AMP-independent mechanism, as that AMPK might act as an “early warning system” involved in antioxidant defense to maintain cellular redox homeostasis [34, 35]. Moreover, Bergheim previously report that AMPK activity does not play a crucial role in steatosis induced by acute alcohol exposure in mice model [36]. Moreover, effects of alcohol on AMPK pathway vary depending on the animal model of ethanol exposure utilized [37]. These results do not preclude a role of AMPK signaling in alcohol-induced liver steatosis but rather suggest that additional pathways may contribute to lipid accumulation. Hence, more studies are needed to assess the functions of AMPK during ethanol-mediated oxidative stress.

PPAR-*α* is a member of the nuclear hormone receptor super family and functions as a lipid sensor in the liver, which is involved in oxidation, transport, and export of free fatty acids [38]. Intracellular concentration of the free fatty acids rises can result in activation of PPAR-*α* by its binding to free fatty acids. Recent results obtained from in vitro and in vivo experiments strongly indicate crucial roles of PPAR-*α* and its downstream target genes in preventing alcoholic fatty liver and injury. In particular, PPAR-*α* modulates expression of several enzymes involved in the mitochondrial and peroxisomal *β*-oxidation of fatty acids [39, 40]. Additionally, It appears that PPAR-*α* deficiency mice were more sensitive to the development of steatohepatitis [41] and PPAR-*α* agonist treatment that prevents steatosis and dampens alcohol-induced hepatic inflammation [42]. In this study, supplement of SGR was shown to significantly upregulate expression of PPAR-*α* in mice, which is expected to accelerate degradation of various fatty acids, resulting in attenuation of alcoholic fatty liver.

**Figure 7:**
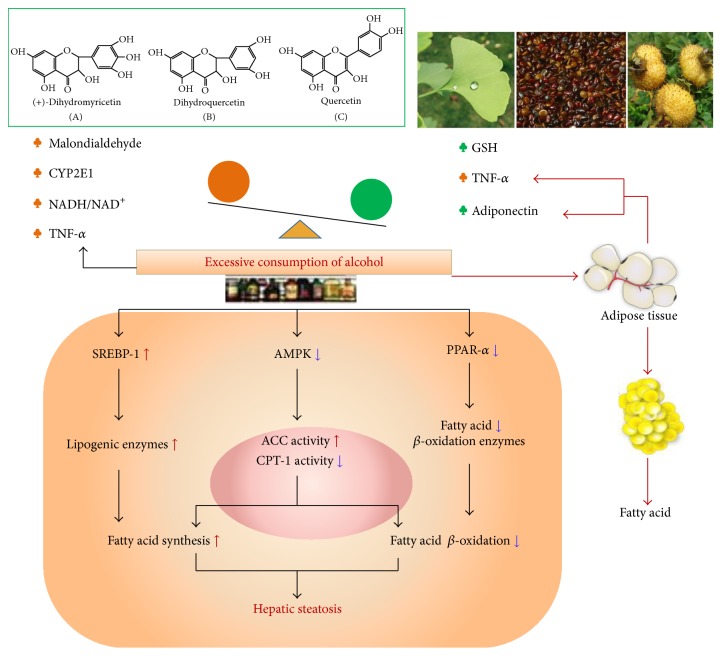
Excessive consumption of alcohol can upregulate the expression of SREBP-1c and downregulate the expression of PPAR-*α*. Meanwhile, alcohol exposure also inhibits AMPK and subsequently increases ACC activity but decreases carnitine palmitoyltransferase 1 (CPT-1) activity. All of them lead to an increase in fatty acid synthesis and a decrease in fatty acid *β*-oxidation. Furthermore, alcohol exposure stimulates adipose tissue adipokine imbalance, such as adiponectin and TNF-*α*. Moreover, alcohol-induced also stimulates adipose tissue lipolysis, the triglycerides reverse transported and deposited in the liver. Moreover, herbal formula SGR contains abundant flavonoids, including dihydromyricetin, dihydroquercetin, and quercetin which inhibit lipogenesis to prevent ethanol-induced liver steatosis.

SREBPs are a family of transcription factors that regulate the enzymes responsible for the synthesis of triglycerides, fatty acids, and cholesterol in liver and other tissues [43]. In particular, the role of SREBP-1c in alcohol-induced fat accumulation has been investigated using in vitro as well as in vivo models and blockage of SREBP-1c was shown to ameliorate fatty liver in ob/ob obese mice [44]. Therefore, ethanol exposure significantly increases the synthesis of the mature SREBP-1c protein which enhances hepatic lipogenesis, thereby leading to the development of fatty liver. In this study, when compared with ethanol animals, our results showed that pretreatment of SGR was shown to significantly downregulate expression of SREBP-1c in mice to attenuate alcoholic fatty liver. Taken together, as shown in Figure 7, the pathogenesis of alcoholic fatty liver is based upon the combination of an increased glycerolipid synthesis and decreased fatty acid oxidation via mitochondrial *β*-oxidation pathway. In this study, the SGR was associated with the decreased TG synthesis and prevention of alcoholic fatty liver in mice, caused by the inhibition of TNF-*α* and mature SREBP-1c, stimulation of PPAR-*α* expression, and AMPK phosphorylation as well as the enhancement of serum adiponectin level. These results indicate that SGR formula prevents acute ethanol-induced liver steatosis via inhibition of lipogenesis and enhancement fatty acid oxidation in mice. Particularly, our findings of this study suggested that acute alcohol exposure elevated the ratio of p-AMPK/AMPK compared to the control group. This phenomenon was brought about by the effects of alcohol on the AMPK pathway that varies depending on the utilized animal model of ethanol exposure, the type of stress applied, and the experimental conditions. Furthermore, acute alcoholic intoxication in present study was a single alcohol exposure which would induce hepatocyte stress response under the physiological condition. ROS induced by acute ethanol ingestion stimulate AMPK activity via an AMP-independent mechanism, as that AMPK might act as an “early warning system” involved in antioxidant defense to maintain cellular redox homeostasis. Hence, more vitro studies are needed to further reveal the hepatoprotective molecular mechanisms of SGR in detail.

## Abbreviations

TNF-*α*: Tumor necrosis factor-*α*
AMPK: Adenosine monophosphate-activated protein kinase
PPAR-*α*: Peroxisome proliferators-activated receptor-*α*
SREBP-1c: Sterol regulatory element binding protein-1c
TG: Triglyceride.
